# Variability in target delineation of cervical carcinoma: A Korean radiation oncology group study (KROG 15-06)

**DOI:** 10.1371/journal.pone.0173476

**Published:** 2017-03-16

**Authors:** Ji Hyeon Joo, Young Seok Kim, Byung Chul Cho, Chi Young Jeong, Won Park, Hak Jae Kim, Won Sup Yoon, Mee Sun Yoon, Ji-Yoon Kim, Jin Hwa Choi, Youngmin Choi, Joo-Young Kim

**Affiliations:** 1 Department of Radiation Oncology, Asan Medical Center, University of Ulsan, College of Medicine, Seoul, Republic of Korea; 2 Department of Radiation Oncology, Samsung Medical Center, Sungkyunkwan University School of Medicine, Seoul, Republic of Korea; 3 Department of Radiation Oncology, Seoul National University Hospital, Seoul, Republic of Korea; 4 Department of Radiation Oncology, Korea University Ansan Hospital, Ansan, Gyeonggi-do, Republic of Korea; 5 Department of Radiation Oncology, Chonnam National University Hwasun Hospital, Chonnam National University Medical School, Hwasun, Jeollanam-do, Republic of Korea; 6 Department of Radiation Oncology, the Catholic University of Korea, College of Medicine, Seoul, Republic of Korea; 7 Department of Radiation Oncology, Chung-Ang University Hospital, Seoul, Republic of Korea; 8 Department of Radiation Oncology, Dong-A University Hospital, Busan, Republic of Korea; 9 Center for Uterine Cancer, Research Institute and Hospital, National Cancer Center, Goyang, Gyeonggi-do, Republic of Korea; North Shore Long Island Jewish Health System, UNITED STATES

## Abstract

**Purpose:**

To determine inter-observer variability in target volume definition of cervical cancer in radical and adjuvant radiotherapy (RT) settings.

**Methods:**

Eight physicians contoured CTVs of 2 patients underwent definitive and postoperative RT. Each volume was analyzed using the individual/median volume ratio and generalized conformity index (CI_gen_). And center of mass (COM) of each contour was calculated. Expert agreement was quantified using an expectation maximization algorithm for Simultaneous Truth and Performance Level Estimation (STAPLE).

**Results:**

For definitive RT, the individual/median volume ratio ranged from 0.51 to 1.41, and CI_gen_ was 0.531. Mean 3-dimensional distances of average to each COM were 7.8 mm. For postoperative RT setting, corresponding values were 0.65–1.38, 0.563, and 5.3 mm. Kappa value of expert agreement was 0.65 and 0.67, respectively. STAPLE estimates of the sensitivity, specificity, and kappa measures of inter-physician agreement were 0.73, 0.98, and 0.65 for the definitive and 0.75, 0.98, and 0.67 for the adjuvant radiotherapy setting. The largest difference was observed in the superior-inferior direction, particularly in the upper vagina and the common iliac area.

**Conclusion:**

As there was still some variability in target delineation, more detailed guidelines for target volume delineation and continuing education would help to reduce this uncertainty.

## Introduction

Despite its declining incidence, uterine cervical carcinoma is the seventh most frequent malignancy and the second most fatal gynecological cancer in Korea, based on recent statistics [1, 2]. Radiotherapy (RT) has long been one of the main treatment options in both curative and adjuvant settings. To reduce treatment-related toxicity by minimizing irradiation of surrounding normal organs without compromising target coverage, intensity modulated radiotherapy (IMRT) has been widely used today [3]. Accurate and reliable definition of the target volume is of the utmost importance to avoid missing the target and to minimize the dose to the surrounding tissue. Consensus guidelines on clinical target volume (CTV) contouring for IMRT of cervical cancer in radical and postoperative RT settings have recently been published [4, 5].

Although these guidelines separated the entire CTV into several components and defined each component using relatively apparent anatomical borders, there is still ambiguity due to (1) anatomical differences among patients (eg, diverse vascular structures, variable degrees of uterus flexion and size), (2) limitations of imaging studies (eg, metal artifacts due to hip replacement), and (3) inter-physician differences (eg, lack of experience, different treatment policies, and level of knowledge). It is unknown how inconsistent CTVs are among experienced radiation oncologists in the era of IMRT in real clinical settings. Therefore, we conducted this study to determine inter-physician variabilities in the delineation of CTVs for cervical cancer in both definitive and postoperative RT scenarios.

## Materials and methods

Experienced radiation oncologists who were Korean Radiation Oncology Group (KROG) members and who specialized in gynecologic malignancies were asked to contour the CTVs of 2 patients treated with primary and postoperative RT using their anonymized computed tomography (CT) simulation image sets. Eight physicians responded and each was provided with the following clinical information: medical history, gynecological examination and blood test results, magnetic resonance imaging (MRI) and positron emission tomography-CT images, and histologic examination results. All clinical information including imaging studies was taken from the hospital which the corresponding author is affiliated with and analyzed anonymously. Since this analysis was performed after completion of the planned treatment of each patient, the patients and physicians involved in current study did not provide written or verbal informed consent prior to participating in the study. However, this study has exempted review as it corresponded to the review exemption condition provided by the institutional review board and approved this non-consent procedure. Patient 1 was a 55-year-old woman diagnosed with stage IIB squamous cell carcinoma who was treated with radical chemoradiotherapy. Pelvic examination revealed a 3.5-cm-sized mass in the uterine cervix with left parametrial invasion. A 5.5-cm-sized mass invading the left parametrium with a left obturator lymphadenopathy was seen in imaging studies. Patient 2 was a 54-year-old woman with stage IB1 disease who underwent radical hysterectomy with bilateral salpingo-oophorectomy and pelvic lymph node dissection. Pathologic examination showed a 2.5-cm-sized adenosquamous cell carcinoma invading the right parametrium without lymph node and surgical resection margin involvement. For simulation, CT images were acquired as a part of routine care, with 2.5-mm slice thickness with intravenous administration of contrast agent using a Light Speed RT instrument (GE Healthcare, St. Giles, UK). Patients were positioned supine, with their arms over the chest. A knee- and ankle-positioning cushion was used for setup reproducibility.

The CTVs obtained by each physician (S1 Fig) were imported from the treatment planning system into MATLAB software (MathWorks, Natick, MA). The agreement in volume determination was quantified by the use of an individual/median volume ratio and a conformity index (CI). The CI was defined as the ratio of the common volume to the encompassing volume. Because the common volume tends to decrease as the number of observers increases, we used a generalized CI (CI_gen_), which is not biased by the number of delineations. The CI_gen_ is defined as:

$${\text{CI}}_{\text{gen}}=\frac{{\sum^{\;}}_{\text{k}=1}^{\text{K}}\text{k}\left(\text{k}-1\right){\text{V}}_{\text{k}}}{2\left(\text{K}-1\right){\sum^{\;}}_{\text{k}=1}^{\text{K}}{\text{kV}}_{\text{k}}-{\sum^{\;}}_{\text{k}=1}^{\text{K}}\text{k}\left(\text{k}-1\right){\text{V}}_{\text{k}}}$$

where k is the number of delineated volumes containing a specific voxel, K is the total number of delineations, and V_k_ is the volume included in k delineations. The k value of each delineated voxel indicates the level of conformity. That is to say, a voxel of k = 1 belongs to a single delineation among all the K observers and therefore its CI value should be zero. In contrast, a voxel of k = K belongs to all the observers’ delineations and thus its CI value should be one. All the delineated voxels can then be binned as a function of k with the associated volume of V_k_, which can be a ‘conformity histogram’. The above formula represents an expectation value of all the different conformity indices of the voxels via the summation over all k values. A detailed derivation of the formula can be found in reference [6]. This expression is simplified into a conceptually meaningful one:

$${\text{CI}}_{gen}=\frac{{\sum^{\;}}_{pairs\;i,j}\left|A_{i}{\cap^{\;}}^{\;}A_{j}\right|}{{\sum^{\;}}_{pairs\;i,j}\left|A_{i}{\cup^{\;}}^{\;}A_{j}\right|}$$

with A_*i*_ and A_*j*_ representing the volumes delineated by the *i*th and *j*th physician, respectively. For spatial comparisons among the 8 contours, the center of mass (COM) of each CTV and its coordinates in right-left (x-axis), anterior-posterior (z-axis), and superior-inferior (y-axis) directions were computed using MATLAB software. Each CTV was then merged into a single image to identify regions that were the most discordant, and inter-physician variability was assessed by calculating differences in 3-dimensional distances, defined as $\sqrt{{\Delta \text{x}}^{2}\;+{\;\Delta \text{y}}^{2}\;+{\;\Delta \text{z}}^{2}}$.

To quantify the agreement among the 8 different CTVs, the expectation maximization algorithm for Simultaneous Truth and Performance Level Estimation (STAPLE) was performed using Computational Environment for Radiotherapy Research (CERR) software [7]. The STAPLE algorithm calculates a probabilistic estimate of a true segmentation from collected delineations, allowing for global analysis and quantification of the performance level of each CTV [8]. This method also enables the development of consensus contours, with various sensitivity, specificity, and agreement level measurements. With CERR software, the following 3 statistics are provided: apparent agreement, kappa-corrected agreement, and STAPLE-estimated probability. The apparent agreement is the probability of an agreement between the observers for each voxel. The kappa-corrected agreement is an agreement value processed to rule out the probability that the agreement could be expected by chance [7]. Generally, it is accepted that a kappa of 0.00–0.20 indicates slight agreement, 0.21–0.40 indicates fair agreement, 0.41–0.60 indicates moderate agreement, 0.61–0.80 indicates substantial agreement, and 0.81–1.00 indicates near perfect agreement. Based on STAPLE analysis, the consensus contours for the CTV for each clinical scenario were generated using the 80% confidence level. Discordant areas between the STAPLE-generated contour and RTOG consensus were reviewed. The maximum distance from the boundaries of the STAPLE volume to those of the contour of each center in 6 directions was computed. These distances were not necessarily in the same plane along the axis.

## Results

The mean volumes of the 8 CTVs were 650 (range, 331–918) mL and 407 (266–562) mL for patients 1 and 2, respectively (Table 1). The maximum CTVs for both patients were obtained by the same physician while the minimum CTVs were obtained by another single physician. The individual/median volume ratio ranged from 0.51 to 1.41, with a standard deviation of 0.33 for radical RT, and the corresponding values for adjuvant RT were 0.65 to 1.38 and 0.24. The CI_gen_ values were 0.531 and 0.563 for patients 1 and 2, respectively, with slightly better agreement in CTV definition in the postoperative RT setting. The mean differences between the average COM and each COM were 1.2 (range, 0.1–3) mm for the right-left direction, 2.7 (0.2–5.1) mm for the anterior-posterior direction, and 6.7 (0.2–19) mm for the superior-inferior direction. Thus, the mean 3-dimensional distance was 7.8 mm for patient 1. For patient 2, the corresponding values were 1.0 (0.5–2.2) mm, 2.7 (0.4–5.3) mm, and 4.2 (0.3–10.4) mm in each direction with a 3-dimensional distance of 7.1 mm. In both patients, the largest difference in the COM was seen in the superior-inferior direction, whereas the right-left direction showed the most consistent COM. More details on CTV analyses were summarized in S1 Table.

**Table 1:** Summary of CTV statistics.

Measure	Patient 1	Patient 2
Number of physicians	8	8
Vol.-Max.	922.28	570.68
Vol.-Min.	341.99	277.20
Vol.-Avg.	657.40	415.36
Vol.-Std.	212.09	99.00
Vol.-Intersection	263.11	167.64
Vol.-Union	1193.67	751.81
Agreement-sensitivity (Avg±SD)	0.73±0.19	0.75±0.12
Agreement-specificity (Avg±SD)	0.98±0.02	0.98±0.02
Overall kappa	0.65-substantial	0.67-substantial
*P* value	< .01	< .01

**Fig 1:**
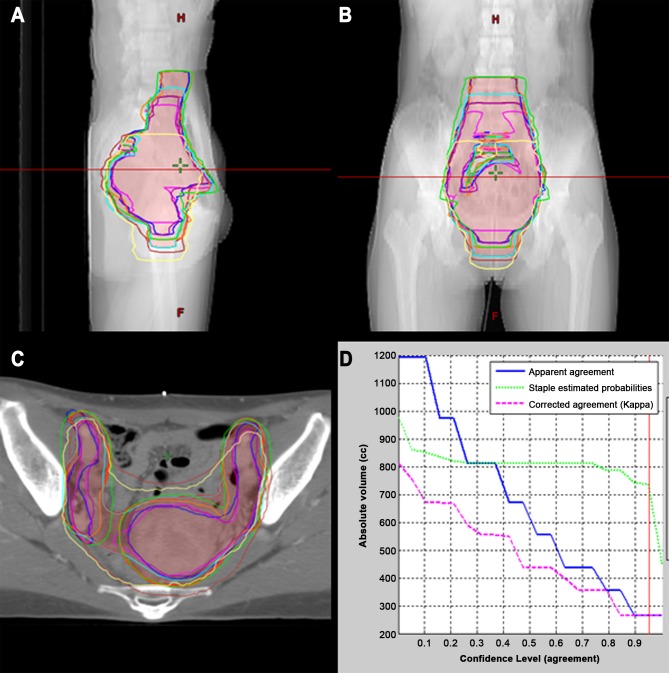
Eight CTVs for patient 1. Axial (A), sagittal (B), and coronal (C) views of all CTVs of the centers. The overlaid STAPLE-generated CTV is drawn in a reddish color. The correlation between the absolute volume (mL) and confidence level (agreement) is shown (D).

**Fig 2:**
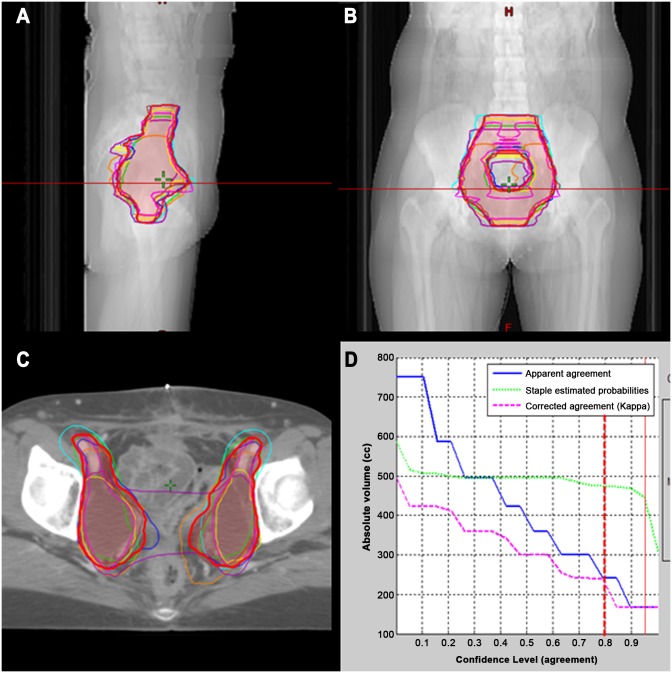
Eight CTVs for patient 2. Patient 2. Axial (A), sagittal (B), and coronal (C) views of all of the CTVs of the centers. The overlaid STAPLE-generated CTV is drawn in a reddish color. The correlation between the absolute volume (mL) and confidence level (agreement) is shown (D).

**Fig 3:**
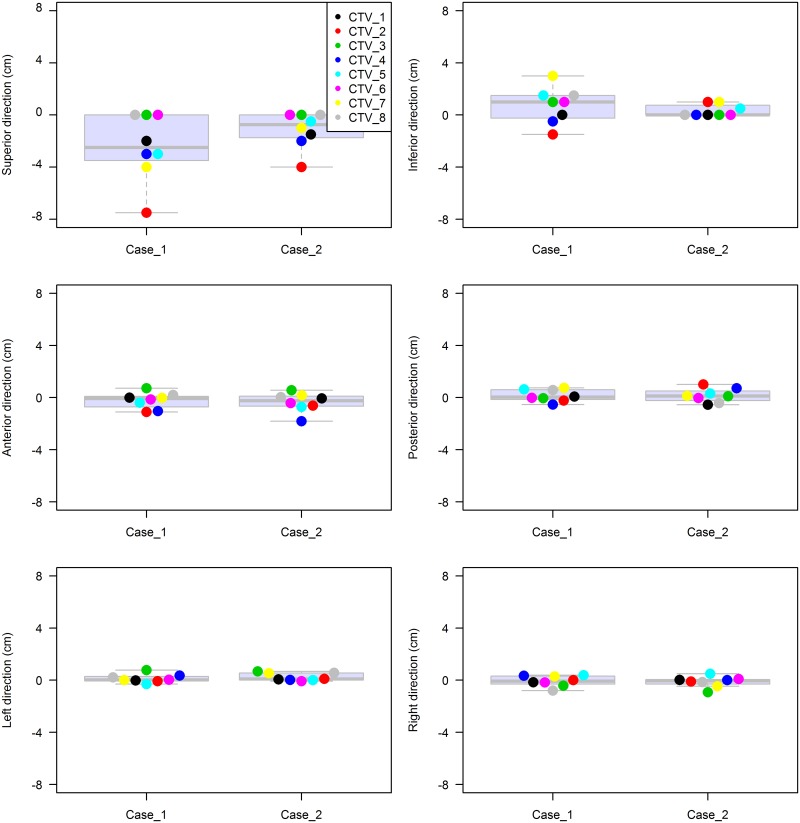
Furthest point along each axis for both patients. Graph illustrating the furthest point along each axis in which the CTV is visible, plotted as the distance from the STAPLE-generated CTV.

STAPLE analysis revealed sensitivities and specificities of 0.73 and 0.98 for curative RT and 0.75 and 0.98 for adjuvant RT, respectively (Table 1). In both patients, CTV delineation showed substantial agreement, with mean kappa values of 0.65 (*P* < .01) and 0.67 (*P* < .01) for patients 1 and 2, respectively. The individual CTV contours and overlaid 80% consensus-generated volumes of patients 1 and 2 in the axial, sagittal, and coronal planes are shown in Figs 1 and 2. The relationship between the absolute CTV and the confidence level (agreement) is also indicated as a reference. The 80% agreement volume generated by the STAPLE algorithm was larger than the mean volume of each center for both patients: the generated volumes were 782.2 and 471.4 mL for patients 1 and 2, respectively. The maximum extension from the STAPLE volume to each CTV was assessed in 6 directions. For patient 1, the largest difference was shown in the superior-inferior axis: 7.5 cm, superiorly, and 3.0 cm, inferiorly. Differences of up to 1–2 cm were observed in the most extreme points along the anterior-posterior and right-left directions. Likewise, the largest variation of 4.0 cm was seen in the superior direction in patient 2. The vagina was more consistently defined, within 1.0 cm, in the adjuvant setting than in the definitive RT setting. In other directions, the difference ranged from 0.7 to 1.8 cm, as shown in Fig 3.

To identify the main component showing discrepancies among contours, we evaluated which anatomic sites of the pelvis were included as part of the CTV using a checkpoint list based on the consensus guidelines [4, 5]. The results are summarized in Table 2. In the definitive RT situation, the inclusion of the common iliac lymph node area was not consistent, which in turn increased the uncertainty at the superior border of each CTV. The treatment volume of the vagina also differed. The maximum difference in vaginal CTVs was 55 mm. Although the variation in the right-left direction was not large, there were differences in the way observers dealt with the surrounding muscles and margin around the vessels. Checkpoint listing results were similar between definitive and postoperative settings. In patient 2, only 2 physicians included the common iliac lymph node area in the CTV, although the guidelines recommend that it be included. Contouring of the external and internal iliac lymph node was consistent. As for the lower border of the CTV, only half of the physicians included the vagina at the level of 3 cm below the vaginal marker. The parametrial/paravaginal tissue was consistently covered, but exclusion of the muscle or bowel loop near the CTV was not done by 3 physicians.

**Table 2:** Checkpoints for consensus guidelines.

		Patient 1	Patient 2
*Nodal CTV*			
7-mm margin around vessels		5	6
Exclusion of muscle		3	5
Exclusion of the bowel		3	5
Common iliac lymph node	Included in the CTV	6	2
	From 7 mm below the L4-5 interspace	4	1
External iliac lymph node	Included in the CTV	8	8
	From the bifurcation to the femoral head level	7	8
Internal iliac lymph node	Along branches (obturator, hypogastric)	8	8
	To the level of the vaginal cuff	6	8
Presacral lymph node	Anterior to the S1 and S2 region	5	6
	Exclusion of the sacral foramen	8	8
Upper vagina^a^	3 cm below the vaginal marker	3	4
Parametrial/paravaginal tissue	0.5–2 cm to the perivesical/perirectal fat	8	8
Lymphocele, surgical clip	Included in the CTV	-	7
Normal organ sparing	Exclusion of the rectum	3	7
	Exclusion of the bladder	3	7

^a^The upper vagina was defined as two-thirds of the upper vagina in patient 1 and 3 cm below the vaginal marker in patient 2.

## Discussion

The results of our present study indicate some variations among participating physicians in CTV definition. A large range in the CTV volume was observed with maximum/minimum volume ratios of 2.8 (patient 1) and 2.6 (patient 2). The maximum and minimum volumes in each patient were obtained by the same physicians. Even if these CTVs were considered outliers and excluded, there would still be a large CTV range, 2.0 and 1.6, respectively. A significant difference in the maximum to minimum volume ratio between 3.6 and 4.9 was reported by a German team [9] before the guidelines were implemented, with a more recent ratio of 1.8 to 2.0 was reported in a study from the UK [10]. The consistency in CTV delineation in our current study was thus better than that of the German study and similar to that of the UK group. We believe that the establishment of the guidelines and expert familiarity with them would at least partly account for the better consistency of recent studies. In our study, the CI_gen_ was 0.531, with a STAPLE-generated sensitivity of 0.73 in the definitive RT setting. For the postoperative setting, the corresponding values were 0.563 and 0.75, respectively, suggesting much room for improvement. Based on the literature, it is generally accepted that CI < 0.5 is considered a poor correlation and CI ≥ 0.7 is acceptable [11]. To investigate the anatomical areas with inconsistent CTVs, we created a checklist containing the following subsites: nodal CTV (common, external, and internal iliac lymph nodes), upper vagina, parametrial/paravaginal tissue, lymphocele, surgical clip, and normal organ sparing. All participating physicians generally followed RTOG guidelines when deciding whether to include or exclude anatomic regions, except for common iliac regions in the postoperative RT setting. Despite the agreement in anatomical components, the size and shape of the CTV varied among the physicians. We attribute this difference to (1) how much margin around vessels was added to make a nodal CTV and (2) whether and how much of the common iliac nodal area was included in the adjuvant RT setting.

As for delineating nodal CTVs for cervical cancer, several studies have shown that pelvic lymph nodes are located adjacent to major blood vessels. Thus, the identification and inclusion of major pelvic blood vessels, with an additional margin of 7–15 mm in general, was the main step in the contouring of nodal CTV. Taylor et al [12] used MRI with intravenous injection of iron oxide particles to identify the location of pelvic lymph nodes in 20 patients. They reported that a 7-mm margin around the vessels encompassed 88% of the pelvic lymph nodes. Chao et al [13] advocated larger margins of 15–20 mm around major vessels and added 15–17 mm medial to the pelvic muscle and bone. Using this larger margin, 100% of the lymph nodes identified in the lymphangiogram were covered. Dinniwell et al [14] defined nodal CTVs in 55 patients with various pelvic malignancies using MRI and the ultra-small super paramagnetic iron oxide contrast agent. A 3-dimensional expansion of a 9-12-mm margin around pelvic vessels and a 12-22-mm expansion anterior to the sacrum and medial to the pelvic side wall were required to encompass most detectable lymph nodes in most patients. According to the RTOG guidelines, at least a 7-mm margin around the vasculature is recommended [4, 5]. However, some of the physicians in our present study used inadequate margins of 2 mm around the vessels. Knowledge of and adherence to the published consensus guidelines on target delineation are strongly encouraged to reduce this uncertainty.

Regarding the common iliac lymph nodal area, most physicians in our present study did not include it in their CTV of patient 2, particularly in the adjuvant setting. It remains unclear whether this area should be covered in the CTV for adjuvant RT, particularly in early-stage cervical cancer patients without any lymph nodal metastasis. Sakuragi et al [15] reviewed surgically treated cervical cancer cases, reporting a positive rate for metastasis to the common iliac lymph node of 9.1% in patients with stage IB to stage IIB. However, when focusing on early-stage squamous cell carcinoma of the cervix (stage I to IIA1), the frequency of common iliac lymph nodal involvement was as low as 1.5% [16]. In an attempt to reduce irradiation of the surrounding normal tissue, small pelvic field irradiation has been investigated. Irradiation of the small bowel and pelvic bone was significantly reduced when the common iliac lymph node was excluded [17], and low acute/late complication rates were subsequently observed [18]. Given that the lymphatic spread of cervical cancer is usually stepwise, the participating radiation oncologists tend to omit common iliac lymph node irradiation in stage IB1 patients without nodal involvement. Further investigation of the RT field of early-stage cervical cancer is warranted in terms of complications and the clinical pattern of failure. The vagina was the next most common organ to show violations of guideline-oriented treatment. It was fully included in both the right-left and anterior-posterior direction but was insufficiently covered inferiorly, as depicted in Fig 2.

This study was not devoid of drawbacks. First, only 2 patients with typical presentations were tested. To discuss various challenging clinical scenarios, more patient data should be analyzed by additional participating experts. Additionally, the knowledge of each expert on the established guidelines and the effects of the adherence of the experts to these guidelines on the consistency of the CTVs were not assessed because the guidelines were published several years before the present study began. Knowledge of and adherence to the established guidelines helped minimize inter-observer variations in trials involving other anatomic sites. For example, the study performed by Mitchell et al [19] showed a significant reduction in inter-physician variability when the guidelines were followed. In that study, 6 radiation oncologists independently delineated the CTV on the CT scans of 3 patients who underwent post-prostatectomy RT. At least 3 weeks later, they defined the CTV again, adhering to the contouring protocol. The maximum/minimum volume ratio in each patient was reduced from 3.7 to 2.0. Lastly, the concept of the internal target volume of the normal organs was not given consideration because we wanted to focus on the CTV. Despite these shortcomings, our present study had unique strengths in that the variability among experts was investigated in several quantitative and qualitative ways in both radical and adjuvant RT settings.

## Conclusion

Although the mean kappa values for the radical and adjuvant RT settings were found to be 0.65 and 0.67, respectively, indicating substantial agreement among experts, there is still some variability in the definition of the CTV for cervical cancer, particularly in the common iliac lymph nodal area and the upper vagina. More detailed and clear guidelines for target volume delineation in conjunction with continuing education might help to further reduce this uncertainty.

## Supporting information

S1 Fig DICOM (Digital Imaging and Communications in Medicine) files of delineated Clinical Target Volume (CTV) by eight experts. (ZIP)

S1 Table CTV analysis in terms of volume, generalized conformity index and center of mass. (XLSX)
